# English-Learning Motivation among Chinese Mature Learners: A Comparative Study of English and Non-English Majors

**DOI:** 10.3390/bs12050135

**Published:** 2022-05-05

**Authors:** Yu Sun, Timothy Teo, Tzu-Hua Wang

**Affiliations:** 1 School of Teacher Education, Shaoxing University, Shaoxing 312000, China; sunyu6336@163.com; 2 School of Education, Murdoch University, Perth, WA 6150, Australia; prof.timothyteo@gmail.com; 3 Department of Education and Learning Technology, National Tsing Hua University, Hsinchu 300193, Taiwan

**Keywords:** Chinese mature learners, English language, English majors, motivation, social network analysis

## Abstract

This study examined the motivation of Chinese mature students toward learning English. The participants in the study were ten Chinese mature learners, of whom four were English majors and six were non-English majors. Each underwent a semi-structured interview. Their responses were then coded and analyzed based on Matsuzaka-Carreira’s motivation framework. Furthermore, the Keyword Extraction and Link Terms techniques of PolyAnalyst^TM^ were used for further analysis. The results showed that English majors tended to be goal-oriented, and non-English majors were more likely to be means-oriented. English majors were identified as having additional integrative motivation. The factors influencing the motivation of English majors were more diverse than those influencing their non-English major counterparts. The obvious distinction between the two groups was their attitude towards the target language and culture. These findings suggest that instruction for adults should be aligned with mature learners’ practical needs and individual characteristics.

## 1. Introduction

For learning English, most researchers and teachers have accepted that motivation is a key factor sustaining effective learning [1]. As learners are motivated to learn in various ways, persistence in English learning can be understood through the analysis of learners’ motivations. Wlodkowskib [2] suggested that an awareness of motives for learning helps teachers to accommodate learners’ needs. In addition to learners who engage in traditional learning, adults may enroll in part-time adult education courses for self-improvement. In explorations of adult learning, motivation is considered a decisive factor affecting mature learners’ enrolment in adult education, and a positive correlation exists between motivation and adult learning [2]. Compared with traditional learners, mature learners are assumed to be more purposeful; they learn English mainly for practical reasons involving work and everyday life, and they value practical knowledge more than they do academic knowledge [3]. In addition to the major effect of practical motives on the learning process, attention should be devoted to social motivation. Social motivation combines with mature learners’ realization of the necessity of adult education in response to social changes [4]. Mature learners’ motivations may therefore be distinguished from those of traditional learners. 

Matures learners’ general motivational orientations have been extensively researched, focusing mainly on the intrinsic and extrinsic dimensions [4,5,6]. Motivational factors and individual experiences have been insufficiently analyzed, with motivation scales and questionnaires the major research instruments. In addition, a few studies have compared the motivations of traditional learners majoring in non-English subjects with those of mature learners [7,8]. It can be seen that the target subjects are non-English majors who occupy a dominant position in the research group, while English majors are scarcely examined. According to Hubackova and Semradova [9], mature learners’ motivations include material factors, professional promotion, and integration into other social groups. Studies using intrinsic and extrinsic dimensions to identify the motivations of mature non-English major learners seem to have incompletely represented the holistic insights attained in adult motivation research fields. Considering the research gaps that currently exist, future studies should use alternative research methods with more motive dimensions for all mature learners.

In the context of mainland China, adult education research has rapidly developed during the last two decades. Adult learners are characterized by social maturity, a stable value system, and tangible practical goals [9]. However, as present teaching strategies do not account for mature learners’ characteristics, English teaching for mature learners in China may be somewhat misaligned with their English learning. Within the body of research on English-learning motivations conducted in China, most researchers have explored university students’ motivation [10,11,12]. With both the number of mature learners and their prominence in social development increasing, adult education has been integrated into many countries’ formal educational systems in order to cater to the needs of mature learners undertaking further study as well as to accommodate their social needs [13]. However, few studies have focused on mature learners, and among these, many have focused on understanding the factors influencing adult learning motivation and strategies for cultivating learning motivation, neglecting the salient effects arising from the differences between English and non-English majors in terms of teaching and learning. This should be considered an important topic given the importance accorded to English education at all levels of the Chinese education system. This study therefore adopted qualitative interviews grounded in the context of mature learners at a Chinese university and investigated and compared the motivation types and motivational factors of English and non-English majors. It is hoped that our findings can help educators to develop teaching methodologies that successfully incorporate mature students’ characteristics in the Chinese context. To achieve this, the following questions are addressed herein:

(1) What are the motivation types exhibited by English and non-English majors among mature learners?
(2) What factors motivate English and non-English majors to learn English?

## 2. Literature Review

### 2.1. Motivation for English Language Learning

The systematic study of language learning motivation was initiated by the Canadian social psychologist Gardner [14] in the context of Canadian bilingualism. His motivation theory built on the idea that language learning occurs in the social milieu where students encounter other cultural communities. In addition to language acquisition and skills development goals, learners within this context are willing to become integrated members of the target language community [14]. This gives rise to two general motivation types, namely, integrative and instrumental motivation. By the 1990s, the theory overwhelmingly dominated discussions on foreign language learning motivation. Hernandez [15] took the integrative and instrumental motivation distinction to be the predictor of achievement in Spanish (as a foreign language) learning for students at a midwestern university in the US. The findings indicated that integrative motivation was more important to successful language acquisition. Hong and Ganapathy [16] conducted a motivation study on students at a Chinese school in Malaysia. Students were found to be more instrumentally motivated than integratively motivated in the English as a second language (ESL) learning context. Furthermore, several researchers have argued that differences might exist between the contexts of English as a second language and English as a foreign language (EFL), as learners in EFL contexts have had insufficient contact with target-language communities. Consequently, several studies have explored differences in motivation within the ESL and EFL contexts [1,17]. However, motivation in language learning is not necessarily a dichotomy between integrative and instrumental motivation. Gardner’s [15] motivation theory was criticized for neglecting educational approaches and failing to adopt multifaceted motivational concepts. Hence, motivational psychology has developed certain cognitive constructs in general educational contexts. One concept that has received language researchers’ attention is self-determination theory, which focuses on the effects of autonomous and controlled motivation. An extensive body of literature has confirmed that autonomous regulation strongly correlates with positive educational outcomes such as higher grades and learning engagement [7,18,19]. The majority of related studies have suggested that a high level of motivation exists where autonomous regulation exceeds controlled motivation [20,21,22]. According to Hayamizu [23], interchangeability may occur between autonomous regulation and controlled motivation. Thus, the viewpoint that autonomous motivation is superior to controlled motivation remains controversial. While motivation theories provide an understanding of learners’ learning activities, motivation within English learning contexts has been insufficiently defined and discussed.

This study thus adopted a motivation framework developed by Matsuzaka-Carreira [24] consisting of eight motivation types from three dimensions: goal oriented versus means oriented, autonomous versus heteronomous, and integrative versus instrumental. As observed in Table 1, despite inevitable overlaps among the eight motivation types, this framework effectively combines motivation theories investigating learners’ learning motivation from various perspectives.

### 2.2. Motivation of Mature English Learners

The investigation of learning motivation mainly takes place in formal education; attention is seldom placed on the field of adult education [25,26,27,28,29]. Recently, adult education has drawn more and more social attention, for the reason that mature learners’ return to education is closely connected to their job, family, environment, and society. Contemporary research on mature learners’ English learning motivation can be classified into three types. The first involves general motivation research, mainly from the intrinsic and extrinsic dimensions [4,5,6]. Carré [4] described the scale of mature learners’ motives to be as follows: from intrinsic perspectives, the epistemic motives are personal enrichment and individual interest, and the socioaffective motives are the establishment of interpersonal relationships; from extrinsic perspectives, the economic motive is salary satisfaction, the vocational motives are the acquisition of professional skills required for professional promotion or competition and pressure or demand from outward expectations, and the social motives are improvement of one’s social status in community events. Second, comparative research has represented an attempt to explore how the motivation of traditional learners with non-English majors differs from that of mature learners’ [7,8]. Generally, mature learners are more intrinsically motivated than traditional learners. This is because their social maturity and life experiences help them to become more autonomous and self-determined during the learning process. Third, few studies have examined the relationship between self-efficacy and self-concept as external variables and motivations [30,31]. The findings suggest that self-efficacy and self-concept positively affect learners’ motivation and learning outcomes. In conclusion, the first two types of research emphasize motivation itself, with limited perspectives from the intrinsic and extrinsic dimensions. The third type is scarce, as other variables gain more prominence in the context of adult education.

Most studies examining mature learners’ motivations have been conducted using a motivation scale. Gardner’s Attitude/Motivation Test Battery [14], which has been widely used in studying language motivation around the world, mainly compares orientations to the second language learning from endogenous and exogenous attribution. Followed by Gardner, Vallerand et al. [32] developed their Academic Motivation Scale, in which motivations related to amotivation were added on the basis of intrinsic and extrinsic dimensions. In addition to the motivation scale, Schmidt et al. [33] designed a questionnaire on motivation using the dichotomy of intrinsic and extrinsic motivation in the context of mature learners learning English as a foreign language. Jacques [34] then improved the questionnaire based on the research of Schmidt et al. [33], with a combination of intrinsic–extrinsic and integrative–instrumental distinctions. The aforementioned motivation scales (questionnaires) were designed either from instrumental and integrative dimensions or intrinsic and extrinsic dimensions. However, more than one motive for learning English can coexist in the same individual. Moreover, these motivation scales or questionnaires were originally designed for investigating traditional learners’ motivation, rather than those of mature learners. Although these scales and questionnaires contribute to examining mature learners’ different motivation types, they do not improve understanding of differentiated motivational factors or individual experiences. In addition, most related studies have focused on examining general motivation orientations, among which non-English majors of mature learners are the target participants, without a full and specific investigation in which English majors are included. Therefore, this study primarily examined the types of motivation that mature learners exhibit using qualitative interviews; additionally, it explored the factors influencing motivation through a comparative study between English and non-English majors.

### 2.3. Mature English Learners in China

In China, English has become a language commonly used for both work purposes and international interactions. The adult education system is more formal, as lots of adults take time for further study after work [3]. Accordingly, the mature learners within this system have unique characteristics. First, they are usually older than university students, with more years spent using mother tongues; this can extent hinder their acquisition of a second language. Second, numerous adult learners have previously failed college entrance examinations or dropped out of school for financial reasons before they subsequently return to adult education, these learners therefore are usually educated to a lower level than university students. Third, mature learners must balance their learning activities with life and work; less time available for learning may affect their learning outcomes [35]. In addition, differences exist between English and non-English majors. Those who choose English as a major mostly work in jobs where the use of English plays a major role; hence, such students have a personal interest at stake, are generally more positive in their learning, and possess a higher level of language ability compared to their non-English major counterparts [36]. Most English majors report that their frequent use of English at work contributes to improving their language competence; by contrast, the majority of non-English majors report that they rarely use English outside of the classroom and have no practical use for it, resulting in a lower level of achievement in English learning [36].

Mature learners are different from university students in terms of their age, social status, and education level [3]. However, current adult teaching has failed to be aligned with the prominent characteristics of these mature learners. At universities, most colleges of adult education share teachers with universities [35]. Because adult teaching is part-time, teachers cannot easily devote adequate attention to this special learner group, and few teachers are willing to study adult learning characteristics or psychology. Textbooks are problematic: regardless of a major’s unique status or situation, and students in the same grade use the same English book (except for English majors) [35]. Furthermore, textbook content is more academic, and is equivalent to the college level [36]. This does not meet mature learners’ learning requirements for practical use, ultimately decreasing their motivation to learn.

## 3. Materials and Methods

### 3.1. Participants

The participants in this study were mature learners (people who did not move on to college directly from high schools) from a university’s college of adult education. The college provided evening courses for adults who worked during the day. Because freshmen were less busy than other students, they were more likely to consent to interviews. Thirty invitation letters were electronically sent to the freshmen, and eighteen of them responded that they were willing to participate in interviews. Ten participants (five females and five males) were ultimately selected in order to provide a balance in majors and genders. Their ages ranged from 22 to 45 years. Among the ten participants, four were English majors; all participants are identified in this paper by the letters A through J, as shown in Table 2.

### 3.2. Instruments

The mature learners who participated in the study were interviewed for 15 to 20 min using QQ, an instant messaging application. The interview questions consisted of two parts. The first established the participants’ attitudes towards English learning and their learning situations, and the second identified mature learners’ motivation types. To verify that the interview questions were appropriate for the subject, comprehensible, and applicable, pilot interviews with two participants were conducted before the study. The views of two university professors from the field of motivation research were obtained as well. Necessary revisions were made according to the provided feedback.

### 3.3. Data Analysis

#### 3.3.1. Content Analysis

Interviews were recorded in Chinese, with the participants’ permission; then, these recordings were recorded in transcripts 2–3 pages in length. The gathered data were analysed using content analysis. After intensive readings, two coders individually divided the obtained data into sections and then coded them. A code list was formed based on their agreements. Finally, the relational codes were compiled according to concept, defined as a stimulus in Matsuzaka-Carreira’s motivation framework [24]. The identification of motivation types was conducted according to these stimuli.

#### 3.3.2. Text Analysis

**Figure 1:**
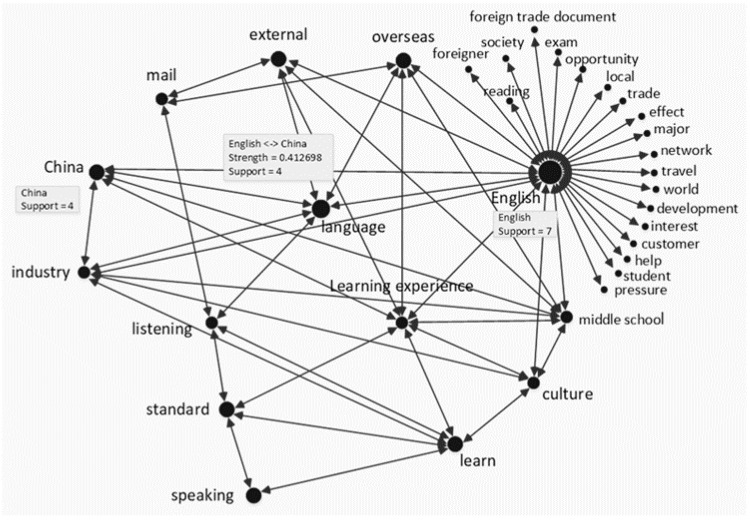
English majors social network analysis.

**Figure 2:**
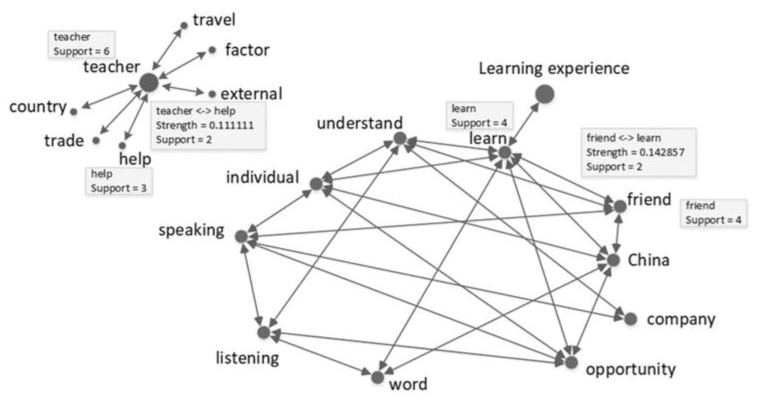
English majors’ social network analysis.

The gathered data were reanalysed using PolyAnalyst ^TM^ text analysis technology (https://www.megaputer.com/polyanalyst/) (accessed on 6 July 2021), which is mainly based on natural language processing. Word were then further analyzed through TF-IDF (term frequency–inverse document frequency) technology. TF-IDF is a statistical method primarily used to calculate the importance of a word and its correlation in a stack of files [37]. First, the interview transcripts (in Chinese) of the English and non-English majors were imported into PolyAnalyst^TM^. Using the Keyword Extraction technique, frequently mentioned keywords in each group’s text were extracted. These keywords described the factors influencing mature learners’ motivations. Then, the Link Terms technique was used to create each group’s keyword correlation. The results of an interactive report displaying each group’s keyword correlation with strength (the probability of the association of one keyword with another, e.g., strength = 0.111) and support (the number of occurrences of a word or the number of co-occurrences between keywords in a sentence, e.g., support = 4) provided insights into how these factors influence English and non-English majors’ English learning. Finally, this was translated into an English version (see Figure 1 and Figure 2).

Note: the dot size of the keyword indicates the frequency of the word’s appearance in the text, that is, the more frequently a word appears, the larger the dot is. The line between two keywords indicates the frequency of two words’ simultaneous appearance in the same sentence. The correlated strength of two keywords can be calculated using the term frequency–inverse document frequency method. The support ‘7’ indicates that the keyword *English* appears seven times in the text. The keyword *English* is correlated with the keyword *China*. The strength ‘0.413’ and the support ‘4’ indicate that the two keywords appear simultaneously four times in the same sentence with a correlation strength of 0.413.

According to the TF-IDF method [37], if the selected records of co-occurrences between two keywords are greater than those of each keyword’s occurrence in a given text, the probability of the co-occurrence of the two keywords will usually be less than 0.5. In addition, as the selected records of co-occurrences between two keywords grows, the probability decreases. In this regard, probability values less than 0.5 are regarded as indicating high correlation between keywords.

## 4. Results

Mature learners’ motivation types were based on four stimuli derived from Matsuzaka-Carreira’s motivation framework [24]: personal desire, external power, utilitarian gains, and integrative reasons.

### 4.1. Motivation Types of English Majors

The data analyzed for English majors show that all of them had positive attitudes towards learning English and were engaged in it. The stimuli presented in Table 3 indicate that these learners were motivated both by job-related pressures, potential promotions or salary increases, an interest in English, a desire to study or work abroad, and a desire to integrate with target-language communities.

According to Matsuzaka-Carreira’s motivation framework [24], a person who desires utilitarian gains and who is absorbed in English learning is described as having goal-autonomy-instrumental motivation, a person learning English for utilitarian gains by external power and who is absorbed in English learning is described as having goal-heteronomy-instrumental motivation, and a person who desires to integrate into target-language communities and who is absorbed in English learning is described as having goal-autonomy-integrative motivation. Responses that English majors provided regarding their motivation types were as follows:

*Participant B: ‘I find learning English a lot of fun. Meanwhile, good language ability to some extent brings me higher salary and is also the requirement of my current job.’*

The responses of participant B indicate a tendency towards goal-autonomy-instrumental motivation and goal-heteronomy-instrumental motivation.

*Participant H: ‘I work hard to learn English not only because of interest or job demands but also for a higher salary to accomplish my desire of studying abroad.’*

The responses of participant H indicate goal-autonomy-instrumental motivation and goal-heteronomy-instrumental motivation, and she had an additional goal-autonomy-integrative motivation.

*Participant I: ‘I like English and I have been working hard for a job promotion that can provide an opportunity to work abroad. This is a good way for me to experience foreign culture and integrate into local life.’*

*Participant J: ‘A better understanding of my foreign customers’ culture and good English proficiency are preconditions for improved communication. Additionally, I work hard to secure my expenses for going abroad to experience life in an English-speaking country.’*

The responses of participants I and J indicate that they have the same motivation types as participant H.

Overall, the English majors could be identified as having goal-autonomy-instrumental, goal-heteronomy-instrumental, and goal-autonomy-integrative motivation for learning English.

### 4.2. Motivation Types of Non-English Majors

The reports of non-English majors indicate that they have negative attitudes towards learning English, and learn English only as a means to an end. The stimuli in Table 4 suggest that English is a means mainly to pursuing job promotions or salary increases, as well as to meeting job requirements.

In Matsuzaka-Carreira’s motivation framework [24], the desire for utilitarian gains and the study of English as merely a means to an end is described as means-autonomy-instrumental motivation; a person learning English for utilitarian gains by external power and studying language merely as a means to an end is described as means-heteronomy-instrumental motivation. The responses that non-English majors have given regarding their motivations are as follows:

*Participant A: ‘I am forced to learn English to better communicate with my foreign customers. I also hope to get an opportunity for job promotion by obtaining an English certificate.’*

*Participant C: ‘Learning English is torture for me, but I have to learn it since my salary is closely related to the amount of goods I sell to those foreign customers.’*

*Participant D: ‘I work hard to improve my English ability in order to be qualified for the position of the overseas sales manager.’*

*Participant E: ‘I have to improve my English language ability so as to deal with lots of overseas orders written in English without mistakes.’*

*Participant F: ‘I’m learning English in preparation for finding a job, because people with good English competency are more likely to get a higher salary.’*

*Participant G: ‘I am made to learn English since I need to introduce our products in English to customers around the world in each year’s international products fair.’*

These reports indicate that non-English majors all possess means-autonomy-instrumental motivation and means-heteronomy-instrumental motivation.

Notably, the two non-English majors’ motivation types changed during the learning process, the evidence for which is as follows:

*Participant D: ‘I had no interest in English at first; I found it interesting after frequent contact with my foreign customers, and gradually had an idea of integrating into the community for further communication.’*

In this regard, the motivation types of participant D changed from means-oriented to goal-oriented. In addition, he exhibited goal-autonomy-integrative motivation.

*Participant E: ‘My foreign customers make me find that English learning is not as boring as I thought before. I really enjoy [discussing] the people, culture, and life in their countries.’*

The responses of participant E reveal that her motivation types became goal oriented, adding goal-autonomy-integrative motivation.

To this end, non-English majors can be identified as having means-autonomy-instrumental motivation and means-heteronomy-instrumental motivation, although their motivation types can change to become goal-oriented and integratively oriented during the learning process (see Table 5).

### 4.3. Social Network Analysis of Factors Influencing English Majors’ Motivation

As detailed in Figure 1, the factors influencing English majors’ motivations are more diverse than those of non-English majors. Social environment, teacher, culture, industry (job), personal interest, and self-improvement are among the factors observed to influence English majors’ learning motivations. Analysis of the participating English majors’ social networks reveals that the keyword *English* is the strongest node, with a strong connection to the keywords *overseas*, *external*, *mail*, *China*, *industry*, *listening*, *standard*, *speaking*, *learn*, *culture*, *middle school*, *learning experience*, and *language*. These keywords are strongly correlated with each other, and have strong strength and support. In addition, the keyword *English* correlates with the other keywords (e.g., *foreigner* and *society*). Although their correlation strength is insufficient, these correlations facilitate the analysis of factors influencing English majors’ motivations.

First, examination of the keyword *external* must take into account that the status of English as the world’s common language due to globalization is agreed upon among English majors. They have an acute awareness of needing to improve their English in response to social needs. The view of one English major reflects this is as follows:

*Participant J: ‘The globalization brings both opportunities and challenges for us. Good English language competence will enhance our competitive power in the workplace. Meanwhile, companies demand greater English proficiency.’*

Second, the keyword *middle school* refers to the prominent role of middle school English teachers in cultivating English majors’ learning interest. The view of one English major reflects this is as follows:

*Participant I: ‘My middle school English teacher made me interested in learning English. He would always design a lot of games to [create an] active classroom atmosphere.’*

Third, the keyword *culture* implies that for English majors understanding the cultural context of the target language is more important than listening, speaking, reading, and writing achievements. This is the reason the majority of English majors wish to integrate into the target-language community. The view of one English major reflects this is as follows:

*Participant H: ‘The way we express ourselves is quite different from that of Westerners, [because of] our different cultural contexts. I think a good cultural understanding can facilitate our language learning.’*

The keywords *speaking, listening, learn, learning experience*, and *China* are considered to be important in the analysis of motivational factors. As shown in Figure 1, the keyword *standard* correlates closely with the keywords *speaking* and *listening*. This indicates that for English majors, *English language standard* refers mainly to listening and speaking language abilities. In addition, English majors exhibit strong self-motivation to improve their language competency. This supports the finding that English majors tend to be goal-oriented in English learning. The keyword *learn* is correlated with the keywords *speaking* and *listening* as well as the keyword *standard*, which implies that English majors have a particular interest in listening and speaking. The keywords *industry* and *culture* are closely connected with the keyword *learn*. This confirms the finding that in addition to goal-autonomy-instrumental motivation, goal-heteronomy-instrumental motivation influences English majors subject to professional pressure and goal-autonomy-integrative motivation arising from cultural interest. The keyword *learning experience* is closely connected with the keyword *English*, as well as being strongly correlated with the keyword *English* and other keywords (e.g., foreigner, society, trade), illustrating that English majors have various learning approaches. One English major’s response reflects this is as follows:

*Participant B: ‘My daily work is mainly to communicate with foreign customers through e-mail. At weekends, I watch some English movies, listen to English songs, and do overseas online shopping. Additionally, I travel abroad sometimes... English has penetrated into each aspect of my daily work and life.’*

Furthermore, the frequent use of the keyword *China* shows how commonly English is used in English majors’ daily lives and work. The correlation between the keyword *China* and the keywords *learning experience*, *language*, and *English* indicates that language learning is part of daily life; the correlation between the keyword *China* and the keyword *industry* indicates language learning is part of the participants’ jobs. English majors have therefore integrated English learning into their daily lives and made full use of various methods to improve their English.

### 4.4. Social Network Analysis of Factors Influencing Non-English Majors’ Motivation

Figure 2 shows that fewer factors influence non-English majors’ motivations compared to English majors. Social environment and industry (job) are the main factors influencing non-English majors’ learning motivation. The keywords *teacher* and *learning experience* are the strongest node belonging to the two clusters. Because no strong correlation exists between the two clusters of keywords, they present two individual patterns of social networks. Notably, although the keyword *learning experience* is one of the strongest nodes, it correlates only with the keyword *learn*. Except for the keyword *learning experience*, all other keywords are strongly correlated within the cluster.

Non-English majors mentioned the keyword *teacher*; in contrast to English majors, non-English majors considered teachers to be unhelpful in their English study. This can be verified by the weakness of the correlation between the keyword *teacher* and the keyword *help* (strength = 0.111). The view of one non-English major reflects this is as follows:

*Participant E: ‘I found learning English was boring since my middle school English teacher just repeated the content of the textbook. Currently … teaching in adult education is also a perfunctory action.’*

In addition, the keyword *teacher* correlates with other keywords. The correlation between the keywords *teacher* and *travel* indicates that traveling facilitates English learning. The responses of non-English majors indicate that the effects of travel are sometimes much more beneficial than those exerted by teachers. The correlations of the keyword *teacher* with the keywords *factor* and *external* imply that teachers are external factors influencing learning achievements. The correlation between the keywords *teacher* and *trade* suggest that non-English majors seek to acquire knowledge of foreign trade from the teacher, and the correlation between the keywords *teacher* and *country* represent the current situation of adult education. One response by a non-English major reflects this is as follows:

*Participant C: ‘I came here in the hope [of obtaining] more practical knowledge on foreign trade, but [the teaching] has not lived up to my expectations. As far as I know, the teachers here are part-time teachers hired from the universities; they don’t care about our learning achievements.’*

The keywords *China*, *learn*, *speaking,* and *listening* appear in non-English majors’ texts. With the exception of the keywords *speaking* and *listening*, all of the non-English majors’ keywords present different information from those of English majors. Analysis of the keyword *China* and its correlation with the keyword *opportunity* indicates that non-English majors realize the necessity of learning English resulting from globalization. However, the difference between English majors and non-English majors in learning English is that English majors are positive learners, while non-English majors are negative about learning. This is aligned with the finding that non-English majors tend to be means-oriented. As they are forced to learn, they use English in class only when necessary. Thus, they discuss their learning experiences only to a limited extent. This might explain why the keyword *learning experience* correlates only with the keyword *learn*. The correlation between the keyword *learn* and the keyword *friend* illustrates that frequent contact with foreign friends helps non-English majors with English learning, and may cause their motivation types to change. The keyword *learn* correlates with the keywords *understand* and *word*, indicating that non-English learners’ language comprehension depends primarily on the understanding of certain keywords. The view of one non-English major reflects this as follows:

*Participant A: ‘I accumulate a number of new words by searching for their meanings in the electronic dictionary; and I often use keywords to communicate with customers in my daily work since people can understand the sentence if they know the meaning of the keywords.’*

## 5. Discussion

### 5.1. Motivation Types of English and Non-English Majors among Mature Learners

This study analyzed Chinese mature learners’ motivation types and the factors influencing their motivations. The research findings show that English majors with goal-oriented (autonomous and heteronomous) motivation are more engaged in learning. This supports Hayamizu’s [23] findings that learners with goal-heteronomy motivation can be absorbed in English learning. By contrast, non-English majors are identified as having means-oriented motivation. Hayamizu [23] states that people with means-oriented motivation regard English learning as a mere means to achieving their goals. The responses of non-English majors indicate that they learn English mainly for higher salaries or for promotions, without any particular learning interest. In addition, Carreira [26] highlighted the comparatively stronger influence of instrumental motivation among adults than among younger children. The reason for this is that mature learners are more purposeful; they learn for practical rather than academic value.

During the learning process, the motivation types of Participants D and E changed from means-oriented to goal-oriented, and both generated additional integrative motivation. Dörnyei’s [1] results indicate that sufficient contact with the target language community produces a high level of integrative motivation that depends on learners’ attitudes and beliefs in the target language community. The responses of these two participants indicated that the changes in their attitudes and motivation to learn English occurred after frequent contact with foreign customers. In addition, Noels [27] stated that integrative motivation might be strongly associated with intrinsic orientation. Accordingly, English majors in the study were identified as having goal-autonomy-integrative motivation. Unlike learners in ESL contexts, they were forced to integrate into the target language community by external factors (e.g., families, teachers, and society) because English is being taught and learned where English is the predominant language of communication. In these circumstances, they might exhibit integrative motivation from an external dimension. However, the statements of the mature learners indicate that their integrative motives result mainly from their interest in the target-language community, thus, their integrative motivations are goal-oriented (autonomy).

### 5.2. Factors Influencing English and Non-English Majors to Learn English

English majors have positive attitudes towards learning English. They are motivated to learn by a desire for self-improvement, affection for teachers, interest in the target culture, utilitarian professional gains, and social needs in response to globalization. They are proficient at making full use of available resources to learn. That is, English learning penetrates every aspect of their daily work and lives. Notably, they are aware that language acquisition should be primarily founded on an understanding of cultural context. Accordingly, Qin and Wen [28] state that the familiarity and mastery of target language culture can enhance learning motivation. The findings explain why English majors have strong goal-oriented motivation to learn English. Moreover, their interest in the target culture and people makes them want to integrate into the target language community. In conclusion, they can be identified as having both integrative and instrumental motivation. This result is in accordance with Al-Oliemat’s [25] findings that English majors are both integratively and instrumentally motivated.

Non-English majors exhibit negative attitudes towards learning English. They learn English mainly for utilitarian professional gains and social needs in response to globalization. Therefore, they are less likely to have integrative motivation because they have no interest in learning English. Suwannatho and Thepsiri [29] similarly concluded that non-English majors have slightly more instrumental motivations than integrative ones. The two special cases of Participants D and E indicate that learning attitudes and motivation types can change during the learning process. It further suggests that this group of mature learners required more contact with target-language communities in order to stimulate their individual interest in English learning.

The motivation types of English majors were identified as goal-oriented and those of non-English majors as means-oriented. This is attributed mainly to learning attitudes; English majors are willing to learn, whereas non-English majors are forced to learn. English majors had more different motives than non-English majors. In addition, English majors showed great interest in the target culture, which significantly influences integrative motivation, such that English majors had additional integrative motivation. Compared with non-English majors, who were externally influenced by social needs and utilitarian gains, the factors influencing English majors’ motivational factors were both internal (self-improvement, interest in target culture, affection for teachers) and external influences (utilitarian gains and social needs). These motivational factors mean that English majors have more diverse learning experiences than non-English majors. English majors use English frequently, both in their daily lives and in their work. According to Liu [31], high-frequency English use helps to improve language competency, and a virtuous language-learning circle results. By contrast, non-English majors seldom use English in their daily lives. Liu [36] states that the infrequent use of English results in a low level of language learning achievement. In this respect, non-English majors’ negative attitudes result in low levels of learning achievement, which in turn sustain negative learning attitudes. This explains why we observed only means-oriented instrumental motivation among non-English majors. This comparative study concludes that attitudes towards target language and culture are major factors influencing the two groups of mature learners’ differing motivation types and learning approaches.

### 5.3. Implications

Because target culture and English teachers are reported to be essential factors influencing learning motivation, the following key implications can be drawn. First, the teaching materials used for mature learners ought to be more practical and closely correlated with the target culture. Second, teachers should design practical learning tasks based on mature learners’ job requirements, as English learners are thereby likely to be more motivated. In summary, adult teaching should provide mature learners with as many opportunities as possible for contact with the target-language community and should devote additional attention to their practical needs.

### 5.4. Limitations and Future Direction

Several limitations should be acknowledged despite the innovative nature of this study. First, the motivation framework used to identify Chinese mature learners’ motivation types are only one of the motivation frameworks containing instrumental and integrative orientations with the four dimensions of goal versus means and autonomy versus heteronomy. We integrated the motivation of mature learners into this framework. Future studies should consider different motivation frameworks for more effective identification of mature learners’ motivation. A secondary consideration is that we examined only motivation types across a comparison of majors. A range of variable individual differences not examined in this study may affect motivation identification. Further work is required to compare mature learners’ age, gender, and work experience. We examined only the current motivation types of mature learners. As mentioned, mature learners’ motivations can change and motivation levels can increase during the learning process. Thus, a long-term study of mature learners’ motivations is required in order to examine more overarching motivation types. Third, although this study sheds light on the English learning motivations of Chinese mature learners, due to constraints on the sample size and interview time, the results cannot be generalized to a broad range of mature learners in mainland China. It is therefore suggested that the sample size be expanded in order to improve the meaningfulness of the results. 

## 6. Conclusions

The present study explored Chinese mature learners’ English-learning motivations. Motivation types were found to differ between English and non-English majors, with English majors tending to be goal-oriented and non-English majors more likely to be means-oriented. Additional integrative motivations were identified, primarily in English majors’ learning processes. English majors’ learning motives originated from internal and external factors; non-English majors were motivated mainly by external factors. The primary difference between the two groups of mature learners in English learning was their attitude towards the target language and culture. Gardner [14] previously confirmed that attitudinal factors significantly affect language learning. In addition, effective teaching that accommodates mature learners’ practical needs and individual characteristics is required. Additional studies on mature learners’ English learning should be conducted as adult education in China continues to develop.

## Figures and Tables

**Table 1:** Matsuzaka-Carreira’s motivation framework for English language learning.

Motivation Types	Definition
Means-autonomy-integrative	Desire for integration into the target language community, with the language only as a means to an end.
Means-autonomy-instrumental	Desire for utilitarian gains, with the target language only as a means to an end.
Goal-autonomy-integrative	Desire for integration into the target language community and absorption in language learning.
Goal-autonomy-instrumental	Desire for utilitarian gains and absorption in language learning.
Means-heteronomy-integrative	Target language learning for integrative reasons by external power, with language learning only as a means to an end.
Means-heteronomy-instrumental	Target language learning for utilitarian gains by external power, with language learning only as a means to an end.
Goal-heteronomy-integrative	Target language learning for integrative reasons by external power with absorption in language learning.
Goal-heteronomy-instrumental	Target language learning for utilitarian gains by external power with absorption in language learning.

**Table 2:** Data related to the participants.

Participants	Gender	Age	Major
A	Male	25	Logistics Management
B	Female	30	English
C	Male	29	Logistics Management
D	Male	26	Administrative Management
E	Female	27	Business Management
F	Female	22	Accounting
G	Male	45	Art Design
H	Female	38	English
I	Female	24	English
J	Male	32	English

**Table 3:** Stimuli of English majors in English learning.

Stimuli	Code	Frequency
Personal desire	Individual interest	4
	Studying/Working abroad	3
External power	Job requirements	4
Utilitarian gains	Higher salary	4
	Job promotion	4
Integrative reasons	Integrate with target language community	3

**Table 4:** Stimuli of non-English majors in English learning.

Stimuli	Code	Frequency
External power	Job requirements	6
Utilitarian gains	Higher salary	6
	Job promotion	6

**Table 5:** Motivation types of English and non-English majors.

Motivation Types		Participants								
	A	B	C	D	E	F	G	H	I	J
Means-autonomy-integrative										
Means-autonomy-instrumental	√		√	√	√	√	√			
Goal-autonomy-integrative				√	√			√	√	√
Goal-autonomy-instrumental		√		√	√			√	√	√
Means-heteronomy-integrative										
Means-heteronomy-instrumental	√		√	√	√	√	√			
Goal-heteronomy-integrative										
Goal-heteronomy-instrumental		√		√	√			√	√	√

## Data Availability

The data presented in this study are available on request from the corresponding author. The data are not publicly available due to privacy.
